# Offline Solid-Phase Extraction and Separation of Mineral Oil Saturated Hydrocarbons and Mineral Oil Aromatic Hydrocarbons in Edible Oils, and Analysis via GC with a Flame Ionization Detector

**DOI:** 10.3390/foods10092026

**Published:** 2021-08-28

**Authors:** José Luis Hidalgo Ruiz, Javier Arrebola Liébanas, José Luis Martínez Vidal, Antonia Garrido Frenich, Roberto Romero González

**Affiliations:** Center for Research in Mediterranean Intensive Agrosystems and Agri-Food Biotechnology (CIAIMBITAL), Department of Chemistry and Physics, Analytical Chemistry Area, Agrifood Campus of International Excellence ceiA3, University of Almería, Carretera de Sacramento s/n, E-04120 Almería, Spain; jhr228@ual.es (J.L.H.R.); arrebola@ual.es (J.A.L.); jlmartin@ual.es (J.L.M.V.); agarrido@ual.es (A.G.F.)

**Keywords:** edible oils, gas chromatography–flame ionization detector, mineral oil aromatic hydrocarbons, mineral oil saturated hydrocarbons, solid-phase extraction

## Abstract

A method was developed for the determination of mineral oil saturated hydrocarbons (MOSH) and mineral oil aromatic hydrocarbons (MOAH) in edible oils, achieving similar limits of quantification than those obtained by online extraction methodologies, i.e., 0.5 mg/kg. The isolation of MOSH and MOAH was performed in a silver nitrated silica gel stationary phase prior to their analysis by gas chromatography–flame ionization detector (GC-FID). To improve the sensitivity, the simulated on-column injection method, using a suitable liner, was optimized. The method was validated at 0.5, 10.0 and 17.9 mg/kg, and recoveries ranged from 80 to 110%. Intra and inter-day precision were evaluated at the same levels, and relative standard deviation (RSD) was lower than 20%. The method was applied to a total of 27 samples of different types of oil previously analyzed in an accredited laboratory, detecting MOSH up to 79.2 mg/kg and MOAH up to 22.4 mg/kg.

## 1. Introduction

Mineral oil hydrocarbons (MOH) are products obtained from the distillation of petroleum and are mainly composed of hydrocarbons, but they are also synthetically produced from coal, natural gas and biomass [1]. These MOH can usually contaminate food in many different ways, by contact with materials that have mineral oils, such as paperboard or inks, mineral oils used in machinery that are utilized during the oil manufacturing process, or even food additives [2]. Lubricating oils for food use are a complex mixture of these aliphatic saturated hydrocarbons and linear or branched ones (paraffin), ranging from C20 to C54 [3].

Moreover, MOH can also be found in edible oil samples that were submitted to a tougher extraction process, such as a second centrifugation of the olive, or to solvent extraction [4]. For this reason, the content of MOH in pomace oils is considerably higher than in other types of olive oils, such as extra virgin olive oil, where the fat extraction is not intense.

MOH can be either mineral oil saturated hydrocarbons (MOSH), which accumulate in tissues, lymph nodes, spleen and liver, and can cause microgranulomas [2], or mineral oil aromatic hydrocarbons (MOAH), which are considered as possible carcinogenic and mutagenic substances [5].

The contamination of edible oils by MOH is becoming a problem of great importance since a severe case of contamination was detected in Ukrainian sunflower oils in 2008, which were contaminated with these compounds at concentrations up to 3100 mg/kg [6]. This fact took the Standing Committee on the Food Chain and Animal Health of the European Commission to set a maximum level of contamination for these compounds, and a limit of 50 mg/kg for crude and refined sunflower oils was established [7].

Oils are always complex matrices to analyze, so several methods were developed to isolate MOH. However, to the best of our knowledge, there is not a method that meets the three following requirements: achieving the separation of both MOSH and MOAH by offline solid-phase extraction (SPE), analyzing them by gas chromatography coupled to a flame ionization detector (GC-FID), and, finally, what can be the most difficult, performing it in vegetable oils. Some studies carried out the offline separation of MOSH and MOAH by SPE, but in different matrices, such as dried foods and cardboard [8,9,10,11], cosmetic lip products [12], or other less fatty matrices, such as pasta, rice, and icing sugar [13], as well as in cereals, chocolate, sausages, and cocoa powder [14]. Moreover, some studies performed the offline extraction by SPE in oils, but they only analyzed the MOSH fraction [15,16,17]. Other studies achieved the goal of separating the MOSH fraction from the MOAH one in oils using a multidimensional chromatographic technique, online liquid chromatography (LC)–GC-FID [14,18,19,20,21,22,23,24], which is more expensive than the offline methodology. In these cases, LC was used to carry out the separation of MOSH and MOAH.

In this study, an offline column chromatography method is proposed to carry out the separation of MOSH and MOAH due to the accuracy and reliability reached, and furthermore, its lower cost and high accessibility by any laboratory, where the use of online methods is not always possible. Moreover, the preparation of the offline chromatographic columns reduces the cost given the much more expensive equipment needed for LC and will achieve the same goal. In conclusion, offline isolation of MOSH from MOAH, and analyzing them via GC-FID in complex samples, such as edible oils, seems to be a good alternative.

Independently of the MOSH/MOAH separation method used prior to the chromatographic determination, the use of FID has widely increased because of its capability in analyzing hydrocarbons and quantifying them according to the number of carbons of the compound; indeed, it is especially used due to the lack of proper analytical standards for the studied compounds and the large number of compounds considered. Therefore, MOSH and MOAH are considered as the sum of several compounds that are chromatographically unresolved, which provide wide humps in the chromatograms.

One of the problems that these methods has is the potential risk of overestimation of the results due to interferences. In this sense, it must be mentioned that olefins usually interfere. Thus, epoxidation can be used to remove them when offline column chromatographic methods are applied [22]. This procedure is simple and quick, improving the selectivity of the method by eliminating interferences. Different epoxidation procedures were developed [20,22], and it was observed that the one proposed by Nestola et al. [22] had several advantages, such as no cooling is necessary and no evaporation of the solvent is needed [25].

Therefore, the development of a sensitive and reliable method that properly separates both fractions (MOSH and MOAH) but at the same time reduces the cost of the analysis is necessary. For this reason, an offline column chromatography coupled to a GC-FID analysis method was developed for the analysis of both fractions. The proposed methodology was successfully tested analyzing 27 samples.

## 2. Materials and Methods

### 2.1. Reagents and Chemicals

The standard mixture of markers and the retention time standard were obtained from Restek (Bellefonte, PA, USA). This standard mixture of markers included (in order of appearance in the chromatogram) *n*-undecane (C11, as a marker for the loss of low molecular mass MOSH), pentylbenzene (5B, as a marker for the loss of low molecular mass MOAH), 1-methylnaphthalnene and 2- methylnaphthalnene (1-MN and 2-MN, as internal standards for the MOSH fraction), bicyclohexyl (CyCy, as the internal standard for the MOAH fraction), *n*-tridecane (C13, present at half concentration as a marker of good sensitivity), 1,3,5-tri-tert-butylbenzene (TBB, as a marker for the beginning of the elution of MOAH), cholestane (CHO, as a marker for the end of MOSH), and perylene (PER, as a marker for the end of MOAH), while the retention time standards included the *n*-alkanes C10, C11, C13, C16, C20, C24, C25, C35, C40, and C50. The standard mixture of *n*-alkanes, ranging from C11 to C40, sodium thiosulfate, and 3-chloroperoxybenzoic acid (*m*CPBA) were purchased from Sigma-Aldrich (St. Louis, MO, USA).

For validation purposes, an oil sample was analyzed by several accredited laboratories by LC-GC-FID, providing a mean concentration of MOSH (38.8 mg/kg) and MOAH (4.2 mg/kg), which were used as the reference values.

LC-MS grade *n*-hexane, toluene, and ethanol were purchased from Honeywell (Morristown, NJ, USA), while ultrapure water was obtained by a gradient system Milli-Q water (Millipore, Bedford, MA, USA). Silver nitrate was provided by Merck (Darmstadt, Germany) whereas dichloromethane and silica gel 60 Å (particle size 0.063–0.200 mm, 70–230 mesh) were purchased from Sigma-Aldrich.

Fritted glass chromatographic columns with a glass stopcock (1 cm of diameter and 20 cm of length) were used, obtained from Pobel (Madrid, Spain).

### 2.2. Instrument and Apparatus

To extract MOH from the samples, a WX vortex from Velp Scientifica (Usmate, Italy) was utilized. An R-114 rotary evaporator from Büchi (Flawil, Switzerland) was used for the evaporation of the solvent.

A Scion GC system equipped with an autosampler (Bruker Corporation, Freemont, CA, USA) was used for chromatographic analyses. An ultra-inert liner SPI 0.25/0.32 mm from Agilent was used to simulate on-column injection. A DB-1HT capillary column (15 m × 0.32 mm i.d. × 0.10 µm film thickness) from Agilent (Santa Clara, CA, USA) was utilized for GC separation after an untreated fused silica capillary column used as pre-column (2 m × 0.32 mm) from Supelco (Bellefonte, PA, USA). The two columns were connected with a press-fit column connection from Agilent. Helium was used as carrier gas at a constant flow rate of 3 mL/min (62.2 cm/s linear velocity). Interactive Graphics (Bruker) v8.2.1 software was used for optimization and quantification.

### 2.3. Sample Collection

The samples were collected from local supermarkets located in Almería (Spain). The total amount of analyzed samples was 27 and they were stored in glass bottles with screw caps in the dark until their analysis.

### 2.4. Preparation of the Chromatographic Columns

The chromatographic columns for the extraction of MOH and isolation of the MOSH and MOAH fractions were prepared manually as no commercial SPE cartridge with 1% AgNO_3_ was available. A 15 mL fritted glass column (20 cm × Φ1 cm) with a glass stopcock was used as the cartridge. The packed sorbent was 6 g of activated silica gel impregnated with 1% silver nitrate. The sorbent was prepared as follows: 100 g of silica gel were weighed out and activated at 600 °C for 6 h in a muffle furnace (JP Selecta, Barcelona, Spain), and then cooled down to room temperature. Consequently, 100 mL of a solution of 1% AgNO_3_ was added drop by drop while shaking into a 1000 mL dark glass bottle. Finally, the sorbent was homogenized on the rotary equipment for 2–3 h and dried in an oven at 125 °C for 12 h. The silica was weekly prepared, and it was maintained in darkness in a dry place at room temperature.

To transfer and pack the sorbent into the column, 12 mL of *n*-hexane were added to the beaker where the silica gel was weighed out. The silica gel with the *n*-hexane was added to the column and then vortexed for 2 min in order to compact the sorbent and let the bubbles go out.

### 2.5. Epoxidation

To remove olefins from the sample, the epoxidation procedure developed by Nestola et al. [22] was used. Briefly, the sample was prepared by exact weighing of 0.6 g of oil and then 1.3 mL of *n*-hexane and 0.05 mL of the solution of internal standards and markers of Restek were added. Then, 1 mL of the ethanolic solution of *m*CPBA (20%) were added and the tube was shaken in a vortex for 10 min. Afterward, 1 mL of ethanol and 4 mL of an aqueous solution of sodium thiosulfate (10%) were added to facilitate the phase separation and eliminate the excess of *m*CPBA. The tube was shaken in a vortex for 30 s and centrifuged at 5000 rpm (4136× *g*) for 5 min.

### 2.6. Extraction

After 40 mL of the mixture *n*-hexane:toluene:dichloromethane (40:40:20, *v/v/v*) and 30 mL of *n*-hexane were added to wash and condition the silica gel, the supernatant (*n*-hexane phase, ~1 mL) phase obtained previously (epoxidation step) was loaded onto the Ag-silica gel column. Then, 8 mL of *n*-hexane were added to collect the MOSH fraction, and 9 mL of *n*-hexane:toluene:dichloromethane (40:40:20, *v/v/v*) were added to collect the MOAH fraction. Finally, 0.3 mL of toluene were added to the MOSH fraction to avoid the total evaporation. The solvents were evaporated in a rotary evaporator with a water bath at 40 °C. It is important to avoid a rapid decrease in pressure and that this pressure does not drop below 200 mbar in the case of MOSH and below 190 mbar in the case of MOAH. The fractions were evaporated until 0.3 mL of toluene was left and they were transferred to a vial with an insert for its injection into the GC-FID instrument.

### 2.7. GC-FID Analysis

In total, 2 µL of the 300 µL of toluene containing the MOSH or MOAH fractions were injected into GC-FID using a liner to simulate on-column injection. The injector temperature was programmed at 100 °C, and directly increased up to 360 °C at a rate of 200 °C/min. Once the temperature was reached, it was held for 10 min. The split valve was closed during the whole analysis.

The oven was at 40 °C and directly increased until 360 °C at a rate of 25 °C/min. Once the maximum temperature was reached, it was held for 15 min. The temperature of the detector was set at 350 °C during the whole analysis. The make-up gas (He), H_2_, and air flow for the detector were 27, 35, and 300 mL/min, respectively.

### 2.8. Integration and Calculation

Integration is a critical part in this study, as it can change the result of the analysis. For this reason, a few instructions must be followed to achieve reliable results.

- A retention time standard mix (C10–C50) must be injected with every set of samples. Thus, the retention times can be properly adjusted.
- The baseline must start at the beginning of the C10 peak and finish at the end of the C50 peak, taking into account the fractions proposed by the JRC guideline (C10–C16, C16–C20, C20–C25, C25–C35, C35–C40, and C40–C50 in the case of MOSH; and C10–C16, C16–C25, C25–C35, and C35–C50 in the case of MOAH) [26].
- Shoulder peaks of the natural hydrocarbons must be subtracted from the hump as they do not belong to the MOH fraction.

To perform the calculations after the integration, Equation (1) was used:

HC = (Area HC × ISTD)/(Area ISTD × Sample),(1)

where “HC” is the concentration of each group of MOSH or MOAH in mg/kg; “Area HC” is the area of the hump subtracting the peaks of the hydrocarbons; “ISTD” is the amount of internal standard in gram; “Area ISTD” is the area of the peak of the internal standard (CyCy for MOSH and 1-MN or 2-MN (the peak with smallest area) for MOAH); and “Sample” is the amount of sample loaded in the chromatographic column in gram.

### 2.9. Method Validation

The JRC guide and the European Commission regulations were used for the validation of the methodology [26]. Trueness in terms of recoveries, intra- and inter-day precision, and limit of quantification (LOQ) per range of hydrocarbons were evaluated.

Linearity was evaluated building a calibration curve diluting the reference oil sample with an extra virgin olive oil blank sample until getting concentrations of 0.5, 1.0, 2.5, 5.0, 10.0, and 17.9 mg/kg for every range of hydrocarbons.

Intra- and inter-day precision and recovery parameters were determined using extra virgin olive oil as a blank and the reference oil diluted with extra virgin olive oil until achieving 0.5, 10.0, and 17.9 mg/kg of MOSH and MOAH.

For intra-day precision, a blank of extra virgin olive oil was spiked five times with the reference oil to have 0.5, 10.0, and 17.9 mg/kg of MOAH and MOAH, and spiked samples were analyzed. For inter-day precision, the same spiking procedure was followed, and the samples were analyzed during five consecutive days.

Trueness was evaluated analyzing five times a reference oil sample analyzed by different laboratories and the error (in %) obtained for the MOSH and MOAH fractions was calculated.

Finally, for the estimation of the LOQ, indications described in the SANTE guideline [27] were followed, defining this parameter as the lowest concentration of the analyte (in this case, it was a group of hydrocarbons of MOH) that was validated with acceptable trueness (recovery ranging from 70 to 120%) and precision (RSD lower than 20%).

## 3. Results and Discussion

### 3.1. Extraction Method Optimization

The olefins that naturally occur in oils enhanced the hump of the MOAH fraction, which led to overestimating the results, because the interfering compounds were taken into account mistakenly; therefore, the quantities were up to ten times larger than they should be. Consequently, the epoxidation described by Nestola et al. [22] was followed, since it considerably improved the results due to the olefins being removed.

An offline column chromatography method was developed. Firstly, 1% of silver nitrate was added to the silica gel due to it is believed that the silver nitrate remained in a crystalline form, filling the pores of the silica gel; thus, the olefins and triglycerides are better retained in the offline column chromatography [15].

Regarding the solvents used to extract the fractions in the chromatographic column, different mixtures were tested. For the MOSH fraction, only *n*-hexane was used as it is a non-polar solvent that dissolves saturated hydrocarbons. Furthermore, it was widely used in previous studies [15,16,17]. In the case of MOAH, different mixtures of solvents were evaluated. It was realized that toluene was necessary for the extraction of all the compounds, as the internal standard PER did not elute if this solvent was not used. According to Kantonales Labor Zurich (Zurich, Switzerland) [28], 20% of dichloromethane should be used, and finally, in the remaining 80%, a mixture of *n*-hexane and toluene (50:50, *v/v*) was added as it was observed that this mixture allowed the elution of the MOAH fraction. Thus, the mixture *n*-hexane:toluene:dichloromethane (40:40:20, *v/v/v*) was selected as the most appropriate for further experiments.

Furthermore, different amounts of sorbent were tested (3.0, 4.0, 5.0, and 6.0 g) to achieve a good separation between MOSH and MOAH. In the same way, the amount of *n*-hexane for MOSH and *n*-hexane:toluene:dichloromethane (40:40:20, *v/v/v*) for MOAH was evaluated analyzing fractions of 1 mL until the adequate markers were observed in the GC-FID. This separation was recognized because of the elution order of the markers. After 8 mL, CHO stopped coming out and the MOSH fraction was considered completely eluted from the chromatographic column. Consequently, TBB started eluting and the MOAH fraction was collected until PER eluted from the column completely after 9 mL.

The washing of the column was also important to minimize the noise as much as possible. Thus, two types of washing procedures were tested: washing the silica in ultrasound or passing a solvent through the column several times. It was checked that the noise did not decrease when the silica was washed in ultrasound. However, when the silica was washed by passing a solvent through the column, the noise was decreasing every 10 mL of solvent until it was checked that 30 mL of the mixture *n*-hexane:toluene:dichloromethane (40:40:20, *v/v/v*) and 40 mL of *n*-hexane were necessary. This solvent washing order was chosen to have in the column the same solvent needed to start the extraction.

The quantity of sample used was also appraised (0.3 and 0.6 g of oil were tested), verifying that 0.6 g of oil was enough to carry out the epoxidation process, having a higher quantity of solvent to transfer the supernatant to the chromatographic column, thus minimizing the risk of collecting the aqueous phase.

Finally, the evaporation conditions were optimized. Different bath temperatures were tested (35, 40, and 45 °C), as well as the minimum vacuum pressures were controlled in order to evaporate the solvents as quickly as possible but avoiding the loss of the most volatile compounds. Thus, in the case of the MOSH fraction, as the solvent was *n*-hexane, the pressure should not be below 200 mbar, while for the MOAH fraction, the minimum pressure should be 190 mbar. When pressures were below these values, the internal standards were lost, as they were low molecular mass compounds, and the analysis was unreliable.

**Figure 1:**
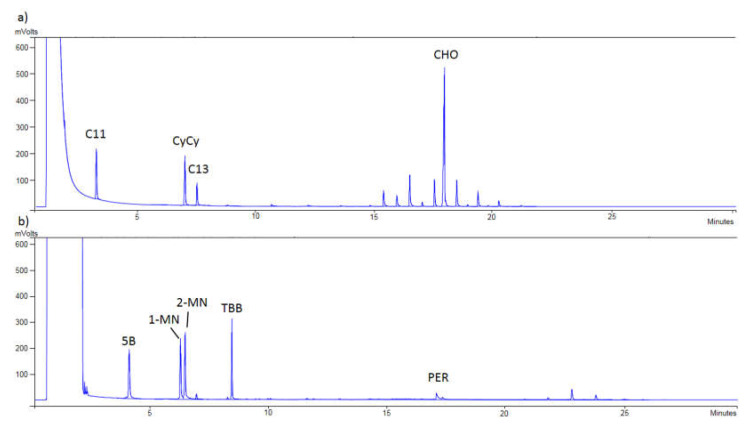
(**a**) Chromatogram of the mineral oil saturated hydrocarbons (MOSH) of an extracted blank sample at 10 mg/kg; (C11: *n*-undecane; CyCy: bicyclohexyl; C13: *n*-tridecane; CHO: cholestane). (**b**) Chromatogram of the mineral oil aromatic hydrocarbons (MOAH) of an extracted blank sample spiked at 10 mg/kg; (5B: pentylbenzene; 1-MN: 1-methylnaphthalene; 2-MN: 2-methylnaphthalene; TBB: 1,3,5-tri-tert-butylbenzene; PER: perylene).

Finally, Figure 1 shows that a proper separation between MOSH and MOAH was achieved, checking the correct appearance of the internal standards in each chromatogram. Thus, in Figure 1a, C11, CyCy, C13, and CHO were detected, while in Figure 1b, 5B, 1-MN, 2-MN, TBB, and PER were monitored.

### 3.2. Optimization of the Chromatographic Conditions

The volatilization of the hydrocarbons can vary depending on several factors, such as the solvent used or the injector and column temperatures. In this study, all the conditions were optimized to obtain the best peaks in terms of peak shape and sensitivity for all the range of the hydrocarbons that are going to be analyzed (C10–C50). For this, 2 μL of a mixture of hydrocarbons at 2 mg/L, ranging from C10 to C50, were injected for on-column simulated injection in order to reduce the amount injected and avoid dirt in the injector. To simulate on-column injection, an ultra-inert liner SPI 0.25/0.32 mm from Agilent was used. Thus, the column could be introduced from one extreme of the liner and got stuck into it, while the syringe was introduced from the other one until the narrowing of the liner, so the sample was introduced directly into the column passing through an “aisle” of the liner. A scheme of this system is shown in Figure S1.

**Figure 2:**
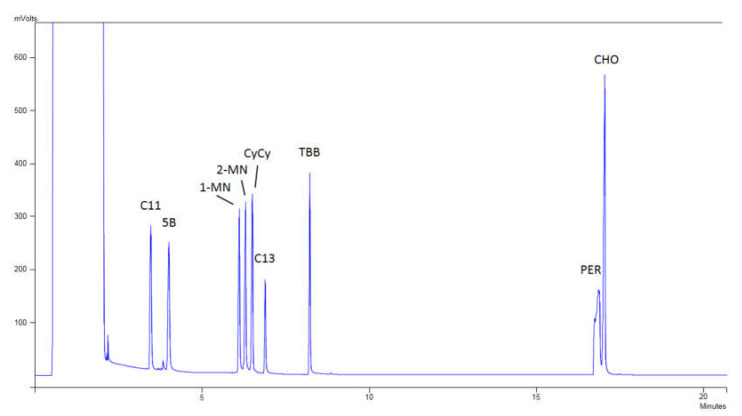
Chromatogram of the mixture of internal standards of Restek spiked at 10 mg/kg in *n*-hexane:toluene (50:50, *v/v*); (C11: *n*-undecane; 5B: pentylbenzene; 1-MN: 1-methylnaphthalene; 2-MN: 2-methylnaphthalene; CyCy: bicyclohexyl; C13: *n*-tridecane; TBB: 1,3,5-tri-tert-butylbenzene; CHO: cholestane; PER: perylene).

The optimization of the injection was carried out in order to lose the minimum amount of volatile compounds and comply with the requirement of a ±20% difference between C20 and C50, as required by the JRC guidelines [26]. Thus, a mixture of hydrocarbons at 2 mg/L ranging from C10 to C50 was injected assaying several minimum (60, 100, and 150 °C) and maximum injection temperatures (340, 350, 360, and 370 °C). Figure S2a shows the results obtained for the minimum temperatures tested in the injector while Figure S2b shows the results obtained for the maximum temperatures. Initially, the solvent was evaporated in the injector at 100 °C and a ramp at 200 °C/min was set. Figure 2 shows a chromatogram of the internal standards of Restek, where it can be observed that the peaks of the compounds are perfectly separated, and no loss of the volatile compounds was produced.

The temperature programming of the oven was also optimized. Five ramps were assayed (50, 35, 25, 20, and 15 °C/min), injecting the linear mixture of hydrocarbons at 2 mg L^−1^. A temperature programming of 25 °C/min provided the correct separation of the lower and higher hydrocarbons. Moreover, the ratio of the lower and higher peaks were tested and the difference was always below 20%, as the JRC guidelines recommend [26].

The temperature of the FID was optimized, injecting the retention time standard at 2 mg L^−1^ at three temperatures (350, 370, and 390 °C). The results showed that at a temperature higher than 350 °C, the noise was higher, and the peak areas did not increase (see Figure S2c), so 350 °C was chosen.

Although MS can also be used [14], this is a more sophisticated detector and less commonly used for this topic.

### 3.3. Method Validation

The evaluation of linearity was carried out through determination coefficients (R2) of the different calibration curves of each range of hydrocarbons from 0.5 to 17.9 mg/kg, depending on the fraction evaluated, and the values were always above 0.99 (see Table S1).

Intra-day and inter-day precisions and recovery values were determined by using an extra virgin olive oil as a blank, previously verified, and the extra virgin olive oil spiked with a reference oil. The average recoveries for MOSH and MOAH are shown in Table 1 and they were between 80.9% and 114.9%. In terms of RSD, the results for the intra-day and inter-day precision were always below 20% (see Table 1). All these parameters are comparable to those obtained in other studies carried out with more sophisticated equipment, such as LC-GC-FID [18,19,20,21].

Trueness was evaluated by analyzing five times a reference oil sample analyzed by different laboratories. The theoretical value for MOSH was 48.8 mg/kg while the total result obtained was 41.0 mg/kg, whereas the theoretical value for MOSH was 4.2 mg/kg and the total result obtained was 5.0 mg/kg. The percentage of error obtained for MOSH was below 16% while for MOAH it was below 19%.

The LOQ was set at 0.5 mg/kg for each range of hydrocarbons of both MOSH and MOAH, as the JRC guidelines require [26]. For this purpose, taking into account the results per range of hydrocarbons of the reference oil, a calibration curve per each range of hydrocarbons was built. This could be performed since the results of the range of hydrocarbons were known individually, as this reference oil was analyzed by several accredited laboratories. This oil measured 1.7, 2.2, 3.2, 17.9, 11.0, and 2.8 mg/kg in the ranges C10–C16, C16–C20, C20–C25, C25–C35, C35–C40, and C40–C50, respectively, for MOSH; and 0.8, 1.0, 1.8, and 0.6 mg/kg in the ranges C10–C16, C16–C25, C25–C35, and C35–C50, respectively, for MOAH. It was verified that an adequate trueness was reached at the lowest point of the calibration curve, 0.5 mg/kg, in each range of hydrocarbons in both MOSH and MOAH, for the setting of the LOQ at this value.

As it can be observed in Table 1, it meets the requirements of the JRC guidelines [26], the recoveries always being between 70 and 120%, and the intra- and inter-day precisions below 20% in terms of RSD. This LOQ value is similar or lower than other studies that used similar approaches (2.5 mg/kg) [16] and is also lower than those that used much complex equipment, such as LC-GC-FID (8 mg/kg) [29].

### 3.4. Sample Analysis

The developed method was applied for the determination of MOSH and MOAH in 27 edible oil samples. A set of internal quality controls (IQC) was injected with every set of samples to guarantee that the analytical procedure was under statistical control. The IQC included a reagent blank, a mixture of the internal standards in a solvent (5, 10, and 20 mg/mL), a mixture of the linear hydrocarbons in a solvent (10 mg/mL), a mineral oil mixture in a solvent (10 mg/mL), and a blank spiked oil sample (10 mg/mL).

Results were reported using the recommended ranges given by the JRC guide [26]. Table 2 sums up the results obtained from the analyzed samples, while in Table S2, the results per range of hydrocarbons can be seen. As can be seen, between C25 and C35, as well as between C35 and C40, the highest concentrations were observed.

As expected, the extra virgin olive oil samples were not contaminated, neither with MOSH nor with MOAH. As can be observed in Table 2, olive pomace oils presented the highest concentrations of MOSH and MOAH, with concentrations up to 79.2 mg/kg of MOSH and 22.4 mg/kg of MOAH. Furthermore, a sample of sunflower oil showed a concentration of MOSH of 15.0 mg/kg and a MOAH concentration of 5.9 mg/kg. Furthermore, the analyzed corn oils were not contaminated.

**Figure 3:**
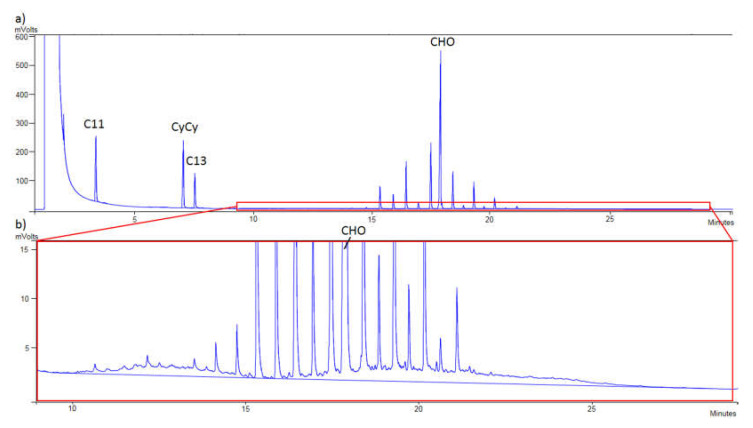
(**a**) Chromatogram of mineral oil saturated hydrocarbons (MOSH) of the sample of refined olive oil 6 contaminated at 8.2 mg/kg; (C11: *n*-undecane; CyCy: bicyclohexyl; C13: *n*-tridecane; CHO: cholestane). (**b**) Zoomed-in from (a) (CHO: cholestane).

**Figure 4:**
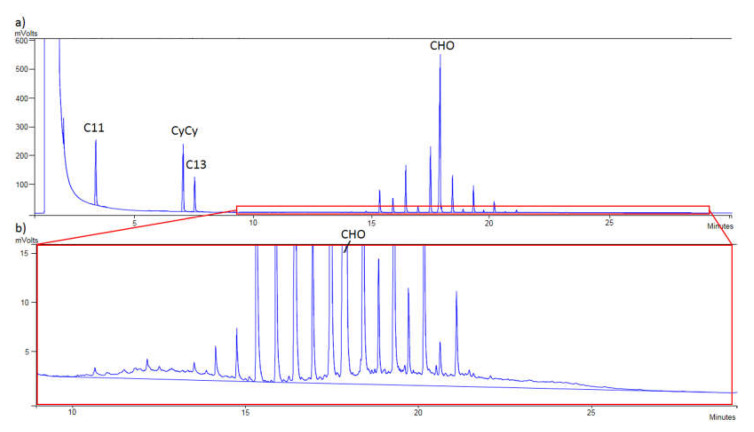
(**a**) Chromatogram of mineral oil aromatic hydrocarbons (MOAH) of the sample of refined olive oil 7 contaminated at 12.9 mg/kg (5B: pentylbenzene; 1-MN: 1-methylnaphthalene; 2-MN: 2-methylnaphthalene; TBB: 1,3,5-tri-tert-butylbenzene; PER: perylene). (**b**) Zoomed in from (a) (PER: perylene).

The sample of refined olive oil 6 contaminated with 8.2 mg/kg of MOSH is shown in Figure 3a while in Figure 3b the same chromatogram can be seen but zoomed in, to appreciate the humps of MOSH. Figure 4a shows the contamination with MOAH (12.9 mg/kg) of the sample of refined olive oil 7, while in Figure 4b, the humps of MOAH can be appreciated as the interesting zone is zoomed in.

Comparing our results with those achieved in other studies, similar results were obtained. For example, Liu et al. [16] found concentrations below 60.9 mg/kg of MOSH in the majority of the samples, except in a blend oil, detecting up to 259.4 mg/kg. Zoccali et al. [19] found low levels of MOSH (below 21.8 mg/kg) but MOAH were not detected in extra virgin olive oils. In addition, they found levels up to 444.8 mg/kg of MOSH and 66.1 mg/kg of MOAH in olive pomace oils.

## 4. Conclusions

A method for the analysis of MOH was developed using an offline extraction followed by GC-FID. The method was fully validated and the LOQ was 0.5 mg/kg for each group of hydrocarbons. The validation of the method also showed suitable trueness and precision values. The validated method was applied to a total of 27 samples of edible oils, detecting MOSH and MOAH in the majority of them. Taking into account that a high amount of MOH was found in the majority of the samples at high concentrations, the routine control of these compounds in edible vegetable oils is necessary.

## Figures and Tables

**Table 1:** Recovery and precision values obtained during the method validation.

Spiked Concentration	Parameter	MOSH	MOAH
0.5 mg/kg	Recovery (%) ^1^	80.9–110.6	105.3–114.9
	Intra-day RSD (%)	15.4	16.4
	Inter-day RSD (%)	18.7	19.3
10.0 mg/kg	Recovery (%)	90.1–105.6	102.3–112.5
	Intra-day RSD (%)	6.4	10.9
	Inter-day RSD (%)	9.7	17.1
17.9 mg/kg	Recovery (%)	92.2–104.3	99.1–108.7
	Intra-day RSD (%)	3.0	6.5
	Inter-day RSD (%)	5.2	8.0

^1^ Recoveries and RSDs were calculated based on *n* = 5.

**Table 2:** Concentration by ranges of MOH detected in the samples of edible oils analyzed.

Matrix	MOSH (mg/kg)	MOAH (mg/kg)
Extra Virgin Olive Oil 1	<LOQ	<LOQ
Extra Virgin Olive Oil 2	<LOQ	<LOQ
Extra Virgin Olive Oil 3	<LOQ	<LOQ
Extra Virgin Olive Oil 4	<LOQ	<LOQ
Extra Virgin Olive Oil 5	<LOQ	<LOQ
Extra Virgin Olive Oil 6	<LOQ	<LOQ
Extra Virgin Olive Oil 7	<LOQ	<LOQ
Refined Olive Oil 1	12.2	<LOQ
Refined Olive Oil 2	5.7	3.3
Refined Olive Oil 3	5.4	5.8
Refined Olive Oil 4	1.1	0.9
Refined Olive Oil 5	<LOQ	<LOQ
Refined Olive Oil 6	8.2	1.4
Refined Olive Oil 7	24.2	12.9
Olive Pomace Oil 1	22.4	7.7
Olive Pomace Oil 2	35.3	19.7
Olive Pomace Oil 3	49.9	18.1
Olive Pomace Oil 4	68.3	21.5
Olive Pomace Oil 5	79.2	22.4
Sunflower Oil 1	<LOQ	<LOQ
Sunflower Oil 2	4.4	<LOQ
Sunflower Oil 3	15.0	5.9
Sunflower Oil 4	<LOQ	<LOQ
Sunflower Oil 5	7.3	3.4
Sunflower Oil 6	<LOQ	<LOQ
Corn Oil 1	<LOQ	<LOQ
Corn Oil 2	<LOQ	<LOQ

## Supplementary Materials

The following are available online at https://www.mdpi.com/article/10.3390/foods10092026/s1, Figure S1: Scheme of the on-column simulation system of injection used, Figure S2: Comparison of the peak areas between the minimum (a) and maximum (b) temperatures of the injector and FID (c) tested. Table S1. Calibration curves and R2 obtained, by ranges, of MOH. Table S2: Concentration by ranges of MOH detected in the samples of edible oils analyzed.
