# Supplementary material for: Offline Solid-Phase Extraction and Separation of Mineral Oil Saturated Hydrocarbons and Mineral Oil Aromatic Hydrocarbons in Edible Oils, and Analysis via GC with a Flame Ionization Detector
Source: Foods. 2021 Aug 28;10(9):2026. doi: 10.3390/foods10092026 (PMC8472750; doi:10.3390/foods10092026)
Supplement: Supplementary file 1 [file foods-10-02026-s001.zip › foods-1340285-supplementary.pdf]

Journal Name: **Foods**

Supporting Information to the paper titled: Off-line solid phase extraction and separation of mineral oil saturated hydrocarbons and mineral oil aromatic hydrocarbons in edible oils, and analysis via GC with flame ionization detector

José L. Hidalgo-Ruiz [1], Javier Arrebola-Liébanas [1], José Luis Martínez Vidal [1], Antonia Garrido Frenich [1] and Roberto Romero González [1,*]

[1] *Department of Chemistry and Physics, Analytical Chemistry Area, University of Almería, Research Centre for Mediterranean Intensive Agrosystems and Agri-Food Biotechnology (CIAIMBITAL), Agrifood Campus of International Excellence ceiA3, Carretera de Sacramento s/n, E-04120 Almería, Spain*

ORCID Codes:

José L. Hidalgo-Ruiz: 0000-0002-8604-2732

Javier Arrebola-Liébanas: 0000-0003-0760-6008

José Luis Martínez Vidal: 0000-0003-0655-2597

Antonia Garrido Frenich: 0000-0002-7904-7842

Roberto Romero-González: 0000-0002-2505-2056

* To whom correspondence should be addressed: R. Romero-González; Tel: +34950214278; Fax: +34950015008. E-mail: rromero@ual.es, University of Almería, Carretera de Sacramento s/n, E-04120 Almería, Spain.

Table of contents

Figure S1. Scheme of the on-column simulation system of injection used.

Figure S2. Comparison of the peak areas between the minimum (a) and maximum (b) temperatures of the injector and FID (c) tested.

Table S1. Calibration curves and R^2 obtained, by ranges, of MOH.

Table S2. Concentration by ranges of MOH detected in the samples of edible oils analyzed.

Figure S1. Scheme of the on-column simulation system of injection used.

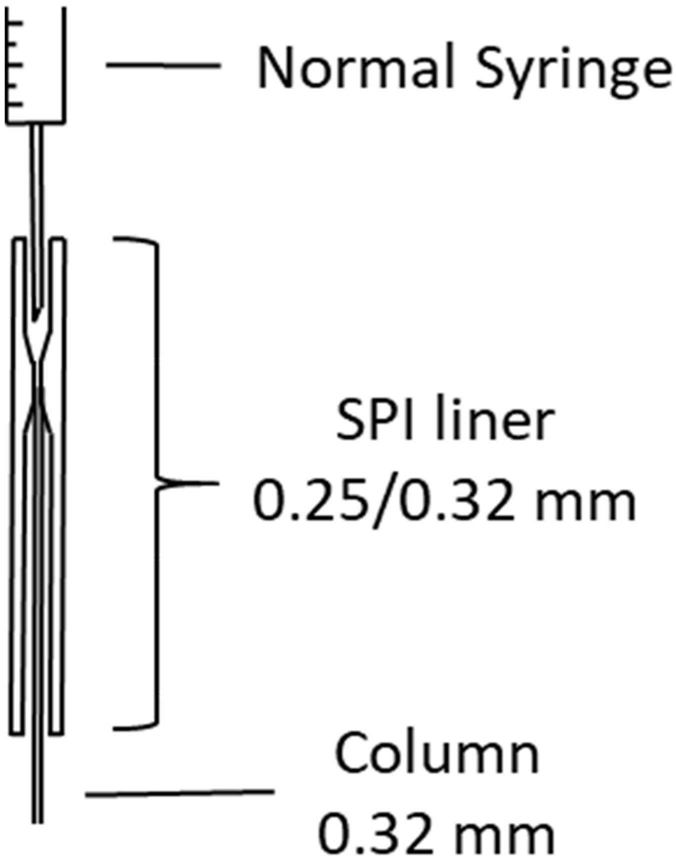

Figure S2. Comparison of the peak areas between the minimum (a) and maximum (b) temperatures of the injector and FID (c) tested.

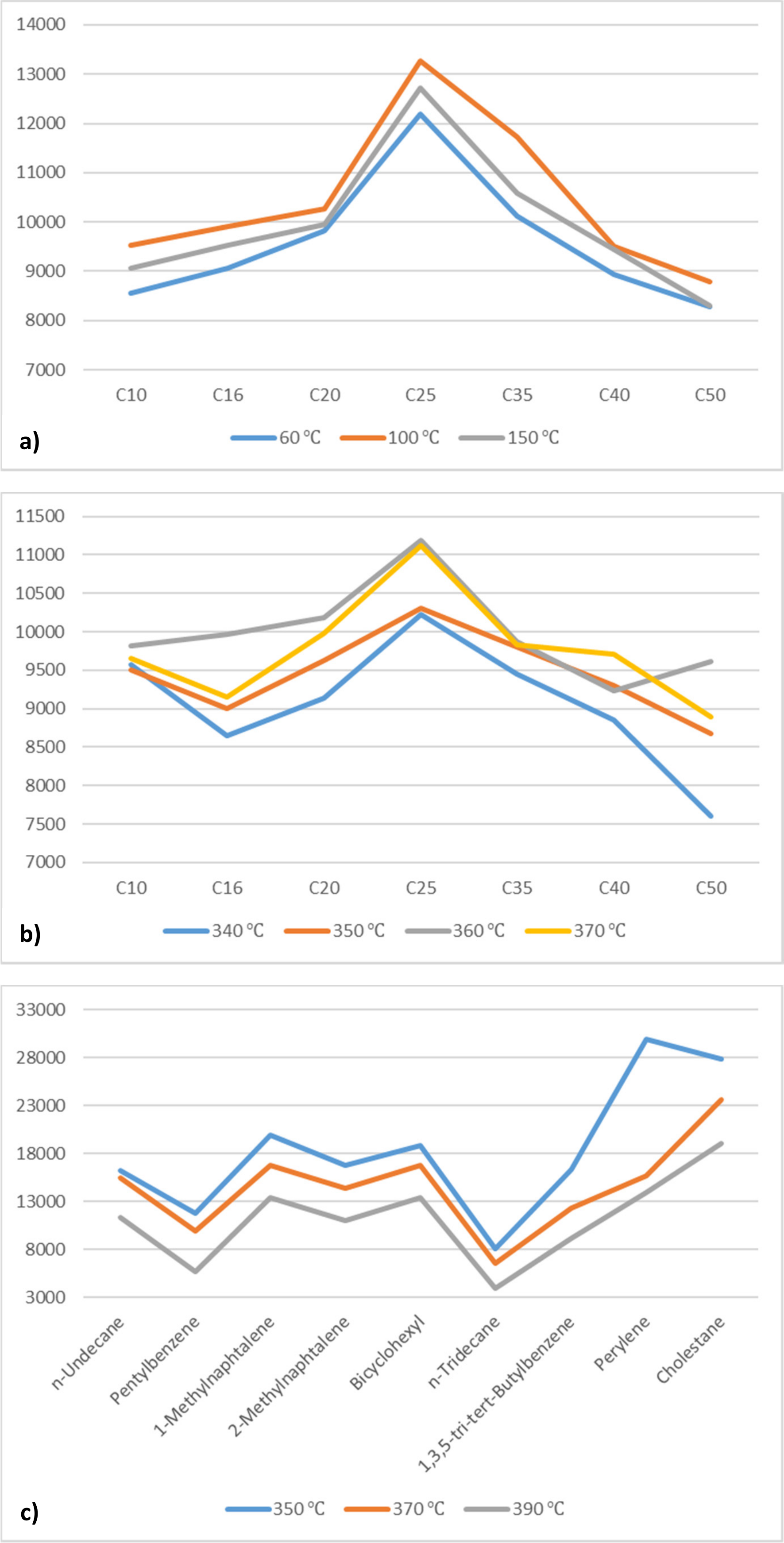

Table S1. Calibration curves and R^2 obtained, by ranges, of MOH.

	Hydrocarbon ranges	Upper Range (mg/kg)	Calibration curves	R^2
MOSH	C10-C16	1.7	y = 1237.7x + 216.49	0.9918
	C16-C20	2.2	y = 1266.3x + 220.97	0.9927
	C20-C25	3.2	y = 1227.1x + 318.73	0.9960
	C25-C35	17.9	y = 1426.9x + 261.92	0.9982
	C35-C40	11.9	y = 1303.4x + 505.37	0.9954
	C40-C50	2.8	y = 1281.1x + 193.66	0.9943
MOAH	C10-C16	0.8	y = 1822.2x + 171.69	0.9909
	C16-C25	1.0	y = 2010.6x + 240.35	0.9920
	C25-C35	1.8	y = 2179.5x + 374.01	0.9933
	C35-C50	0.6	y = 1432.4x + 200.60	0.9935

Table S2. Concentration, by ranges, of MOH detected in the samples of analyzed edible oils.

	MOSH (mg/kg)							MOAH (mg/kg)				
	C10-C16	C16-C20	C20-C25	C25-C35	C35-C40	C40-C50	**Total**	C10-C16	C16-C25	C25-C35	C35-C50	**Total**
Extra Virgin Olive Oil 1	<LOQ	<LOQ	<LOQ	<LOQ	<LOQ	<LOQ	**<LOQ**	<LOQ	<LOQ	<LOQ	<LOQ	**<LOQ**
Extra Virgin Olive Oil 2	<LOQ	<LOQ	<LOQ	<LOQ	<LOQ	<LOQ	**<LOQ**	<LOQ	<LOQ	<LOQ	<LOQ	**<LOQ**
Extra Virgin Olive Oil 3	<LOQ	<LOQ	<LOQ	<LOQ	<LOQ	<LOQ	**<LOQ**	<LOQ	<LOQ	<LOQ	<LOQ	**<LOQ**
Extra Virgin Olive Oil 4	<LOQ	<LOQ	<LOQ	<LOQ	<LOQ	<LOQ	**<LOQ**	<LOQ	<LOQ	<LOQ	<LOQ	**<LOQ**
Extra Virgin Olive Oil 5	<LOQ	<LOQ	<LOQ	<LOQ	<LOQ	<LOQ	**<LOQ**	<LOQ	<LOQ	<LOQ	<LOQ	**<LOQ**
Extra Virgin Olive Oil 6	<LOQ	<LOQ	<LOQ	<LOQ	<LOQ	<LOQ	**<LOQ**	<LOQ	<LOQ	<LOQ	<LOQ	**<LOQ**
Extra Virgin Olive Oil 7	<LOQ	<LOQ	<LOQ	<LOQ	<LOQ	<LOQ	**<LOQ**	<LOQ	<LOQ	<LOQ	<LOQ	**<LOQ**
Refined Olive Oil 1	<LOQ	<LOQ	1.1	9.8	1.3	<LOQ	**12.2**	<LOQ	<LOQ	<LOQ	<LOQ	**<LOQ**
Refined Olive Oil 2	<LOQ	<LOQ	<LOQ	3.2	2.5	<LOQ	**5.7**	<LOQ	<LOQ	3.3	<LOQ	**3.3**
Refined Olive Oil 3	<LOQ	<LOQ	<LOQ	4.1	1.3	<LOQ	**5.4**	<LOQ	<LOQ	4.5	1.3	**5.8**
Refined Olive Oil 4	<LOQ	<LOQ	<LOQ	1.1	<LOQ	<LOQ	**1.1**	<LOQ	<LOQ	0.9	<LOQ	**0.9**
Refined Olive Oil 5	<LOQ	<LOQ	<LOQ	<LOQ	<LOQ	<LOQ	**<LOQ**	<LOQ	<LOQ	<LOQ	<LOQ	**<LOQ**
Refined Olive Oil 6	<LOQ	<LOQ	<LOQ	6.0	2.2	<LOQ	**8.2**	<LOQ	<LOQ	1.4	<LOQ	**1.4**
Refined Olive Oil 7	<LOQ	<LOQ	2.2	19.0	3.0	<LOQ	**24.2**	<LOQ	<LOQ	7.4	5.5	**12.9**
Olive Pomace Oil 1	<LOQ	<LOQ	1.1	15.1	6.2	<LOQ	**22.4**	<LOQ	2.5	5.2	<LOQ	**7.7**
Olive Pomace Oil 2	<LOQ	<LOQ	1.0	12.3	11.1	10.9	**35.3**	<LOQ	4.0	15.0	0.7	**19.7**
Olive Pomace Oil 3	<LOQ	1.2	1.7	18.5	16.4	12.1	**49.9**	0.9	2.5	14.7	<LOQ	**18.1**
Olive Pomace Oil 4	0.5	1.4	5.0	25.0	22.3	14.1	**68.3**	<LOQ	2.1	17.4	2.0	**21.5**
Olive Pomace Oil 5	0.6	1.1	<LOQ	30.7	24.6	22.2	**79.2**	0.8	1.9	19.7	<LOQ	**22.4**
Sunflower Oil 1	<LOQ	<LOQ	<LOQ	<LOQ	<LOQ	<LOQ	**<LOQ**	<LOQ	<LOQ	<LOQ	<LOQ	**<LOQ**
Sunflower Oil 2	<LOQ	<LOQ	<LOQ	3.1	1.3	<LOQ	**4.4**	<LOQ	<LOQ	<LOQ	<LOQ	**<LOQ**
Sunflower Oil 3	<LOQ	<LOQ	<LOQ	12.0	3.0	<LOQ	**15.0**	<LOQ	<LOQ	5.1	0.8	**5.9**
Sunflower Oil 4	<LOQ	<LOQ	<LOQ	<LOQ	<LOQ	<LOQ	**<LOQ**	<LOQ	<LOQ	<LOQ	<LOQ	**<LOQ**
Sunflower Oil 5	<LOQ	<LOQ	<LOQ	4.8	2.5	<LOQ	**7.3**	<LOQ	<LOQ	2.2	1.2	**3.4**
Sunflower Oil 6	<LOQ	<LOQ	<LOQ	<LOQ	<LOQ	<LOQ	**<LOQ**	<LOQ	<LOQ	<LOQ	<LOQ	**<LOQ**
Corn Oil 1	<LOQ	<LOQ	<LOQ	<LOQ	<LOQ	<LOQ	**<LOQ**	<LOQ	<LOQ	<LOQ	<LOQ	**<LOQ**
Corn Oil 2	<LOQ	<LOQ	<LOQ	<LOQ	<LOQ	<LOQ	**<LOQ**	<LOQ	<LOQ	<LOQ	<LOQ	**<LOQ**
